# Reliability and Validity of the Lithuanian Version of CASP-19: A Quality of Life Questionnaire for the Elderly

**DOI:** 10.3390/medicina54060103

**Published:** 2018-12-05

**Authors:** Andrejus Černovas, Vidmantas Alekna, Marija Tamulaitienė, Rimantas Stukas

**Affiliations:** Faculty of Medicine of Vilnius University, 03101 Vilnius, Lithuania; vidmantas.alekna@mf.vu.lt (V.A.); marija.tamulaitiene@mf.vu.lt (M.T.); rimantas.stukas@mf.vu.lt (R.S.)

**Keywords:** psychometric properties, quality of life, elderly

## Abstract

*Introduction:* Ageing is associated with several physical, psychological, and behavioral changes. These changes are closely related with general health problems and quality of life in old age. The CASP-19 multidimensional instrument was specially designed to measure quality of life in the elderly. The different language versions of this scale have been used in more than 20 countries. However, Lithuanian translation was not available. The objective of our study was to test psychometric properties of the Lithuanian version of the CASP-19 questionnaire. *Materials and methods:* A cross-sectional study was performed with ambulatory men and women aged 60 and older, living in a community in Vilnius, Lithuania. Exclusion criteria were current acute illness, malignant tumor, and Mini-Mental State Examination (MMSE) score < 25 points. Psychometric properties of CASP-19 were tested using reliability and validity methods. *Results:* The study sample consisted of 132 participants, 28 (21.8%) of them were men and 103 (78.2%) women. Analysis of psychometric properties of the Lithuanian version of CASP-19 showed high internal consistency (Cronbach’s alpha 0.85), good agreement between test-retest measures with an ICC of 0.82 (95% CI 0.79–0.85) and good convergent and divergent construct validity. *Conclusions:* The psychometric properties indicated that the Lithuanian version of CASP-19 was reliable and valid. As such, it might be used to evaluate quality of life in elderly people.

## 1. Introduction

One of the negative consequences of the ageing of the global population is the increase in health problems and illnesses, which may soon overwhelm health systems in all countries. Quality of life (QoL) in later life has become a research target and global policy issue [1]. QoL is a multidimensional concept that covers many health components and life domains [2]. Ageing society is a global phenomenon that requires preservation of a good QoL and promotion of successful ageing in later life. According to Rowe and Kahn [3], successful ageing has three components: active engagement with life, high mental and physical function, and avoiding disease and disability. All these components will influence QoL in old age [4,5,6,7]. Typically, universal (or non-specific) questionnaires were used to evaluate the QoL—the European Quality of Life (EuroQol) five dimensions–adult version questionnaire (EQ-5D), developed by a network of international, multilingual, multidisciplinary researchers (The EuroQol Group) [8], and the Medical Outcomes Study 36-Item Short Form Survey Instrument (SF-36) [6] produced by the Survey Research Group at the RAND Corporation (Santa Monica, California, USA).

The CASP-19 multidimensional instrument was designed to measure a quality of life in the elderly. The 19 item questionnaire is composed of four domains, and the first initial of each domain makes up the acronym—Control, Autonomy, Self-realization, and Pleasure [9]. These four domains cover a theory of measuring needs satisfaction [10]. Doyal and Gough describe control as the ability to shape one’s own life situation and environment, while autonomy refers to self-determination or an absence of unwanted interference from others. Self-realization is defined as the ability to identify goals and initiate actions to achieve them, and is identified as a basic human need [11]. The pleasure domain deals with aspects of well-being and sense of worth. Laslett defines pleasure and self-realization domains as the pursuit of enjoyable activities which satisfy in later life when child-rearing and career development aims have been met [12].

The CASP-19 has been used in more than 20 national and international studies. It has already been translated into 16 languages. This tool has been included in the Survey of Health, Aging, and Retirement in Europe (SHARE) [13], the English Longitudinal Study of Ageing (ELSA) [14], the Health, Alcohol, and Psychosocial factors in Eastern Europe study (HAPIEE) [15], the Health and Retirement Survey (HRS) [16], and the Irish Longitudinal Study of Ageing (TILDA) [17]. A review of the cross-national validation studies shows that CASP-19 is well-developed. There are a number of different factor models and item variations of the measure, which include versions that are 3-factor, 12-item (UK population, 2008) [18], 2-factor, 9-item (Irish population, 2013) [17], 5-factor, 19-item (Taiwanese population, 2013) [19], 4-factor, 16-item (Brazilian population, 2014) [20], 4-factor, 11-item (Ethiopian population, 2015) [21], 5-factor, 19-item (Malaysian population, 2017) [22], and 3-factor, 12-item (Spanish population, 2018) [23]. However, Lithuanian translation had not been initiated until now. There were no tools to measure the quality of life in elderly persons or manage international projects with the possibility of comparing results with others countries.

The aim of our study was to test psychometric properties of the Lithuanian version of the CASP-19 questionnaire.

## 2. Materials and Methods

### 2.1. The CASP-19 Questionnaire

The CASP-19 questionnaire consisted of 19 items. For each of these items, participants were asked to rate how often a statement described how they felt, for example, “My age prevents me from doing the things I would like to do,” or, “I enjoy the things I do.” Using a 4-point Likert scale, a participant would respond with one of the following options: often = 0, sometimes = 1, rarely = 2, or never = 3. Thirteen positively worded items (3, 5, 7, 10–19) were reverse-coded. The total CASP-19 score ranged from 0 to 57; higher scores indicated higher QoL. The CASP-19 was a self-administered questionnaire, and could be completed in approximately 10–15 min [9].

### 2.2. Lithuanian Translation and Cross-Cultural Adaptarion of CASP-19 Questionnaire

The Lithuanian translation of CASP-19 was performed according to guidelines for the process of cross-cultural adaptation of self-report measures [24] and the World Health Organization guidelines [25] According to these guidelines, five stages were followed:

*Stage* *1:* *Initial translation.* An initial translation from English to Lithuanian was performed by two independent bilingual translators, both of whom were native Lithuanian speakers. Translator 1 had a medical background and knowledge about health care terminology. Translator 2 had no knowledge about medical terminology or the construct of the instrument.

*Stage* *2:* *Synthesis of the translations.* A consolidation of these two translations in which a consensus was reached and a preliminary initial translated version of the instrument in the target language was generated.

*Stage* *3:* *Backward translations.* The preliminary initial translated version was backward translated into the original language by two more independent bilingual translators, both native English speakers with no medical background or knowledge about the original English version of the CASP-19 questionnaire.

*Stage* *4:* *Expert committee review.* The expert committee included one methodologist (the investigator of a research team who is familiar with content areas of the construct of the instrument), one linguistic specialist, and four translators who had performed translation and back-translation. The expert committee compared two back-translations with the original questionnaire and reviewed the grammatical structure of the sentences and similarity in meanings. Expert committee members whose mother language was Lithuanian enhanced the quality of the pre-final version of the translated instrument. Any ambiguities and discrepancies regarding cultural meaning and colloquialisms or idioms in words and sentences of the questionnaire were discussed and resolved through consensus of a pre-final version of the questionnaire.

*Stage* *5:* *Test of pre-final version.* Pre-testing of the CASP-19 pre-final version was performed to ensure good comprehension of each question. Fifteen individuals were asked to rate items using a dichotomous scale (clear or unclear) and to provide suggestions as to how to rewrite the statements to make the language clearer. Any obscurity was discussed and resolved by the expert committee, and the final version of the Lithuanian CASP-19 was conducted.

### 2.3. Validation and Psychometric Properties of CASP-19 Questionnaire

*Study* *sample.* The cross-sectional study was performed on ambulatory men and women aged 60 and older. Exclusion criteria were: mother language is not Lithuanian, Mini-Mental State Examination (MMSE) < 25 points, present acute illness, or malignant tumor. The elderly participants were recruited to the study by a convenience sampling method, after which interviews and meetings were scheduled with the researchers.

*Internal* *consistency* is an important measurement property for questionnaires that intend to measure a single underlying concept (construct) by using multiple items. It was evaluated by Cronbach’s alpha coefficient.

*Construct* *validity.* In addition to the CASP-19 questionnaire, another QoL measure—EQ-5D—was also completed. The EQ-5D consisted of five dimensions titled mobility, self-care, usual activities, pain/discomfort, and anxiety/depression, and each dimension had three levels—no problems, moderate problems, or extreme problems. The patient indicated health state by ticking the box next to the most appropriate statement in each of the five dimensions. This decision resulted in a one-digit number that expressed the level selected for that dimension. The digits for the five dimensions were combined into a five-digit number that described the patient’s health state [8]. Construct validity was assessed using convergent and divergent validity. Correlation analysis was performed between CASP-19 and EQ-5D total scores and separate domains of EQ-5D that covered similar dimensions.

*Test-rest* *reliability.* We received the second CASP-19 questionnaire from 34 participants, but after exclusion of two persons with more than 20% missing data and two persons whose health had changed between the administrations, we obtained a sample of 30 subjects for the evaluation of the test-retest reliability.

*Floor* *and* *ceiling* *effects* were defined as when a high percentage of the participants had the lowest or the highest score, respectively. If floor or ceiling effects were present, it meant that some items may have been missed in the lower or upper end of the scale, possibly limiting content validity.

### 2.4. Compliance with Ethical Standards

All subjects gave their informed consent for inclusion before they participated in the study. The study was conducted in accordance with the Declaration of Helsinki, and the protocol was approved by Vilnius Regional Biomedical Research Ethics Committee (3 August 2016; No:158200-16-828-346).

### 2.5. Statistical Analysis

A Shapiro–Wilk test was used to determine normality of quantitative variables. Variables were reported as mean ± standard deviation for normally distributed data, and as median with interquartile ranges (IQR) for non-normally distributed data. The presence of significant differences between the two groups (men and women) was calculated using Student’s t-test and Mann-Whitney’s U-test for quantitative variables without normal distribution. The questionnaire’s internal consistency and homogeneity was evaluated by Cronbach’s alpha coefficient. A value between 0.70–0.90 indicated a good internal consistency. A value lower than <0.70 was considered questionable, and >0.90 indicated there were redundancies in the questionnaire [26]. Pearson or Spearman correlations were used to test construct validity (convergent and divergent) of the questionnaire depending on the distribution of the variables. A correlation of less than 0.20 was considered insufficient, between 0.21 and 0.40 as acceptable, 0.41 and 0.60 as good, 0.61 to 0.80 as very good, and >0.80 as excellent. Test-retest reliability was measured using the Intraclass Correlation Coefficient (ICC) and its confidence interval (CI) at 95%, with the CASP-19 questionnaire considered reliable as an acceptable reliability with ICC value >0.70 [26]. Floor and ceiling effects were defined as when a high percentage of the participants had the lowest or the highest score, respectively. A floor or ceiling effect of ≥15% was considered significant [27].

The statistical analysis was performed using IBM SPSS Statistics 18.0 software. Results were considered statistically significant based on probability, *p* < 0.05.

## 3. Results

The study sample consisted of 132 participants with an average age of 73.1 ± 8.1 years and was comprised of 28 (21.8%) men and 103 (78.2%) women. Most participants (72.7%) were living in an urban area. Analyzing education level, we found that 89 (59.8%) were well educated, 14 (10.6%) had finished vocational or professional school, 26 (19.7%) had a post-secondary or special secondary school education, and 13 (9.9%) had finished secondary school. Of the participants, 56 (42.4%) were widowed, 21 (15.9%) had never married, 19 (14.4%) were separated/divorced, and 36 (27.3%) were still married. Detailed scores from the CASP-19 and EQ-5D questionnaires for men and women are shown in Table 1.

According to the Lithuanian Statistics department database [28], the participants closely matched the demographics of the Vilnius district population by gender and living area. Most of the participants were well educated and widowed. Mean income of the participants was 340.55 ± 171.48 Euro per month.

Shapiro-Wilk normality testing revealed that CASP-19 results were normally distributed. The potential range of CASP-19 was from 0, which represented “a complete absence of quality of life” to 57, which represented “total satisfaction of control, autonomy, self-realization and pleasure domains.” The range of scores in this study was 12–57. The scale exhibited a slightly negative kurtosis with a mean of 35.8, a median of 37, and a standard deviation of 10.2. No participant scored zero and only two participants achieved the highest possible score of 57; therefore, floor and ceiling effects were not significant.

Table 2 shows mean, inter-item correlations, and internal consistency for each of CASP-19 items.

General Cronbach’s alpha coefficient was 0.852, indicating a high level of internal consistency. The inter-item correlations ranged from 0.065 to 0.56, with the weakest area observed for question A6, “Family responsibilities prevent me from doing what I want to do”. Among participants who completed a second CASP-19 questionnaire after an interval of two weeks, we found a good agreement level between test-retest measures with an ICC of 0.82 (95% CI 0.79–0.85).

The correlation matrix of total scores and the four domains of CASP-19 was assessed using Pearson’s correlation coefficient (Table 3).

Correlation coefficients ranged from 0.39 to 0.84. The CASP-19 total score correlated strongly with each of the domains—control, autonomy, self-realization, and pleasure. The strongest correlation was between CASP-19 total score and the self-realization domain score, while the weakest was between autonomy and self-realization domain scores.

Results of construct validity of CASP-19 when compared with EQ-5D questionnaires are shown in Table 4.

Data of the EQ-5D questionnaire were not normally distributed; therefore, we used Spearman’s correlation method to evaluate correlations between the total score of CASP-19 and the individual domains scores of the EQ-5D questionnaire. We found moderate correlations between the CASP-19 total score and the scores of the EQ-5D index domain (*r* = 0.39, *p* < 0.001), the scores of the EQ-5D mobility domain (*r* = −0.37, *p* < 0.001), and the EQ-5D usual activities domain (*r* = −0.36, *p* < 0.001). We found negative correlations between the CASP-19 total score and the EQ-5D self-care domain (*r* = −0.32, *p* = 0.04), the EQ-5D pain/discomfort domain (*r* = −0.17, *p* = 0.04), and the EQ-5D anxiety/depression domain (*r* = −0.3, *p* = 0.001). Based on these results, the CASP-19 questionnaire was a reliable and valid instrument which could be used for clinical practice and research purposes.

## 4. Discussion

This study evaluated a Lithuanian version of CASP-19 which, after translation and cultural adaptation, has been found to be a reliable and valid tool for assessing QoL in the elderly. Because the originally CASP-19 had been developed and validated in English, this study aimed to provide a Lithuanian version of the questionnaire and test its psychometric properties. The Lithuanian version of the CASP-19 questionnaire showed high internal consistency, construct validity and a good test-retest reliability. These results were similar to those found in other studies [9,15,17,29].

The mean score for CASP-19 in this study was 35.8 ± 10.2, compared to 42.2 ± 7 that Hyde et al. [9] reported in their first report on CASP-19; an additional study reports scores of 42.5 ± 8.7 [14]. Sim and colleagues returned scores of 40.2 ± 9 in an Irish population [29]. All of these CASP-19 scores were higher than our results. These differences may be related to a different socioeconomic status and health care system in Great Britain and Ireland.

We have found that the CASP-19 mean scores were lower in men than women (31.8 ± 10.1 and 36.8 ± 1, respectively). According to results from the Health, Alcohol, and Psychosocial factors in Eastern Europe (HAPIEE) study [15], there were significant differences in mean values for CASP-19 between participants in the Czech Republic, Russia, and Poland (*p* < 0.001). Men scored significantly higher on CASP-19 than women in all countries: Czech Republic (men 35.5 ± 9.9; women 34.3 ± 10.2), Russia (men 34.5 ± 9.3; women 33.1 ± 8.5), and Poland (men 38.0 ± 9.3; women 36.8 ± 8.9). Polish men and women reported the highest CASP-19 scores. The lowest CASP-19 scores were reported for Russian men, a score similar to what we found with Lithuanian women. However, the total CASP-19 score of Lithuanian men was the lowest. This could be explained by the different life expectancies for men and women in Lithuania; in 2016, life expectancy was 69.49 years for men and 80 years for women [30]. The construct validity analysis of our results showed that the total CASP-19 scores were strongly and statistically correlated with the individual control, autonomy, self-realization, and pleasure domains. The CASP-19 scores showed a weak correlation with EQ-5D mobility and EQ-5D usual activities domain scores, indicating convergent validity of CASP-19. Moreover, weak correlations were found between CASP-19 and EQ-5D self-care, pain/discomfort, and anxiety/depression domains, confirming that CASP-19 was divergent with domains expected to be divergent. Previous studies have found evidence of construct validity of the CASP-19 questionnaire. Sim et al. [29] reported a moderately high (*r* = 0.66) correlation between CASP-19 and Satisfied With Life Scale (SWLS) scores, but lower correlations between CASP-19 and SF-12 physical and mental components (*r* = 0.53 and *r* = 0.49). According of Bowling and Stenner [31], based on data from three national surveys of the elderly in Great Britain, CASP-19 scores correlated with the scores from the Older People’s Quality of Life (OPQoL) questionnaire in the Ethnibus (*r* = 0.49) and the Office of National Statistics (ONS) national Omnibus (*r* = 0.74) survey data. Correlation between CASP-19 and World Health Organization Quality of Life (WHOQoL-OLD) assessment scores were almost the same in the Ethnibus survey (*r* = 0.38) and in the ONS Omnibus survey (*r* = 0.69). The HAPIEE study results showed correlations between CASP-19 and Center for Epidemiologic Studies Depression Scale (CES-D) scores—Czech Republic population (*r* = −0.49), Russian population (*r* = −0.40), and Polish population (*r* = −0.57). Correlation between CASP-19 and SF-10 scores were the same in Czech Republic and Russia (*r* = 0.40), and slightly higher in Poland (*r* = 0.41) [15].

The limitations of our study were that the CASP-19 questionnaire was tested only on citizens of Vilnius district and that the study sample was rather small. Results could be different when citizens of other regions of Lithuania are surveyed.

## 5. Conclusions

The analysis of the psychometric properties of the Lithuanian version of the CASP-19 questionnaire indicates that it is reliable and valid. The Lithuanian version of the CASP-19 questionnaire may be used to evaluate quality of life in elderly people.

## Figures and Tables

**Table 1:** Scores of CASP-19 and EQ-5D quality of life questionnaires in men and women.

Variable	Score			
	Total	Men	Women	*p* Value
CASP-19, mean ± SD	35.76 ± 10.22	31.79 ± 10.05	36.83 ± 10.04	0.02 *
Control domain, mean ± SD	6.7 ± 3	5.11 ± 3.35	7.13 ± 2.76	<0.001 *
Autonomy domain, mean ± SD	9.67 ± 2.95	8.93 ± 2.85	9.87 ± 2.96	0.13 *
Self-realization domain, mean ± SD	10.57 ± 3.29	9.71 ± 3.17	10.80 ± 3.30	0.12 *
Pleasure domain, mean ± SD	8.82 ± 3.66	8.04 ± 3.84	9.03 ± 3.60	0.23 *
EQ-5D, median (IQR)	0.8 (0.7–0.9)	0.8 (0.68–1.0)	0.8 (0.73–0.85)	0.85 **

CASP-19—Control, Autonomy, Self-realization, Pleasure 19-items questionnaire; EQ-5D—European Quality of life 5 Dimensions; SD—standard deviation; IQR—Interquartile range; *p* value: for difference in scores between men and women; * Student’s t-test; ** Mann—Whitney U test.

**Table 2:** Internal consistency for each item in CASP-19 questionnaire.

Domain	Items	Mean	Std. Deviation	Corrected Item-Total Correlation	Squared Multiple Correlation	Cronbach’s Alpha If Item Deleted
Control	C1	1.61	0.95	0.426	0.557	0.861
	C2	1.52	1.02	0.419	0.347	0.861
	C3	1.89	1.07	0.540	0.473	0.856
	C4	1.68	1.07	0.541	0.449	0.856
Autonomy	A5	2.14	0.1	0.545	0.474	0.856
	A6	2.33	0.97	0.065	0.305	0.874
	A7	2.01	0.98	0.414	0.429	0.861
	A8	1.55	1.02	0.543	0.532	0.856
	A9	1.64	1.07	0.161	0.352	0.872
Self-realization	S10	2.21	0.97	0.390	0.329	0.862
	S11	2.11	1.01	0.561	0.530	0.856
	S12	2.20	0.88	0.426	0.557	0.861
	S13	2.00	0.90	0.419	0.347	0.861
	S14	2.04	0.92	0.540	0.473	0.856
Pleasure	P15	1.86	0.97	0.541	0.449	0.856
	P16	1.23	1.00	0.545	0.474	0.856
	P17	1.98	1.05	0.065	0.305	0.874
	P18	2.14	0.93	0.414	0.429	0.861
	P19	1.61	1.07	0.543	0.532	0.856

**Table 3:** Correlation coefficients for CASP-19 questionnaire and its four domains.

Scores	Total	Control	Autonomy	Self-Realization	Pleasure
Total	1.000				
Control	0.766 *	1.000			
Autonomy	0.729 *	0.554 *	1.000		
Self-realization	0.819 *	0.464 *	0.386 *	1.000	
Pleasure	0.840 *	0.455 *	0.429 *	0.696 *	1.000

CASP-19—Control, Autonomy, Self-realization, Pleasure 19-items questionnaire; * *p* < 0.001.

**Table 4:** Construct validity of CASP-19 score.

	CASP-19 Total Score, *r* Value	*p* Value
Convergent validity		
EQ-5D index	0.39	<0.001
EQ-5D: mobility	−0.37	<0.001
EQ-5D: usual activities	−0.36	0.03
Divergent validity		
EQ-5D: self-care	−0.32	0.04
EQ-5D: pain/discomfort	−0.17	0.04
EQ-5D: anxiety/depression	−0.3	0.001

CASP-19—Control, Autonomy, Self-realization, Pleasure 19-items questionnaire; EQ-5D—European Quality of life 5 Dimensions questionnaire.
